# Characterization of the in vitro metabolic profile of nazartinib in HLMs using UPLC-MS/MS method: In silico metabolic lability and DEREK structural alerts screening using StarDrop software

**DOI:** 10.1016/j.heliyon.2024.e34109

**Published:** 2024-07-05

**Authors:** Mohamed W. Attwa, Ali S. Abdelhameed, Adnan A. Kadi

**Affiliations:** Department of Pharmaceutical Chemistry, College of Pharmacy, King Saud University, Riyadh, Saudi Arabia

**Keywords:** Nazartinib, Greenness, Metabolic stability, StarDrop software, DEREK program, UPLC-MS/MS

## Abstract

The orally given, irreversible, third-generation inhibitor of the epidermal growth factor receptor (EGFR), known as Nazartinib (EGF816), is now undergoing investigation in Phase II clinical trials conducted by Novartis for Non-Small Cell Lung Cancer. The primary aim of the current research was to establish a rapid, specific, environmentally friendly, and highly versatile UPLC-MS/MS methodology for the determination of nazartinib (NZT) levels in human liver microsomes (HLMs). Subsequently, same approach was used to examine the metabolic stability of NZT. The UPLC-MS/MS method employed in HLMs was validated as stated in the bioanalytical method validation criteria outlined by the US- FDA. The evaluation of the metabolic stability of NZT and the identification of potentially structural alarms were performed using the StarDrop software package that includes the P450 and DEREK software. The calibration curve for NZT showed a linearity in the range from 1 to 3000 ng/mL. The inter-day accuracy and precision exhibited a range of values between −4.33 % and 4.43 %, whereas the intra-day accuracy and precision shown a range of values between −2.78 % and 7.10 %. The sensitivity of the developed approach was verified through the determination of a LLOQ of 0.39 ng/mL. The intrinsic clearance and in vitro half-life of NZT were assessed to be 46.48 mL/min/kg and 17.44 min, respectively. In our preceding inquiry, we have effectively discerned the bioactivation center, denoted by the carbon atom between the unsaturated conjugated system and aliphatic linear tertiary amine. In the context of computational software, making minor adjustments or substituting the dimethylamino-butenoyl moiety throughout the drug design process may increase the metabolic stability and safety properties of new synthesized derivatives. The efficiency of utilizing different in silico software approaches to conserve resources and reduce effort was proved by the outcomes attained from in vitro incubation experiments and the use of NZT in silico software.

## Highlights

• The current manuscript is considered the first analytical study for NZT quantification in HLMs.
• The metabolic stability of NZT was esti8mated using in vitro and in silico tools.
• DEREK software was used to screen for possible structural alerts.
• Structural modifications to the dimethylamino-butenoyl may improve the metabolic stability of NZT new derivatives.

## Graphical abstract

**Figure:**
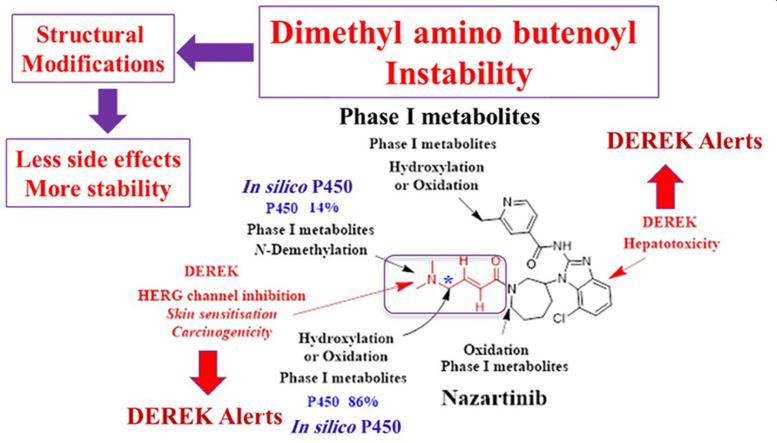

## 1 Introduction

Lung cancer currently has the uppermost fatality frequency among various types of cancers in developed regions, including Europe and North America. There are two primary types of lung cancer, namely small-cell lung cancer (SCLC) and non-small-cell lung cancer (NSCLC) [1]. According to the World Health Organization (WHO), the international mortality rate attributed to lung cancer in 2018 exceeded 1.76 million fatalities. The frequency of its manifestation is steadily increasing. According to a comprehensive evaluation conducted in 2018, the global incidence of lung cancer reached a significant figure of 2.1 million newly diagnosed cases, representing approximately 11.6 % of the total international cancer burden. NSCLC encompasses approximately 90 % of all diagnosed lung cancer cases, exhibiting several subtypes that arise from the activation of various oncogenes [2,3]. Current therapeutic approaches in cancer management rely on the molecular targeting of tumor suppressor oncogenes and genes, that have an important role in the development of the disease in human beings [4]. Despite the relatively sluggish progress in the discovery of novel cancer treatments, recent advancements in molecular targeting strategies have unveiled significant improvements in patient prognosis [5]. Nevertheless, groundbreaking medical interventions that emerged in the early 2000s, including immune checkpoint inhibitors and targeted therapy, have notably prolonged the lifespan of individuals afflicted with metastatic NSCLC [6,7]. Health authorities in multiple countries have authorized the use of targeted treatments to combat fusions including ALK, ROS1, EGFR, NTRK, V600E, and BRAF. In addition, there is ongoing active research and development of therapeutic interventions aimed at addressing abnormalities in EGFR, RET, exon 20 insertions, KRAS G12C, HER2, and MET. In May 2020, the USFDA granted approval to selpercatinib as the initial RET inhibitor and capmatinib as the inaugural MET inhibitor.

NSCLC is a diverse collection of lung cancer subtypes that impacts a substantial number (about 90 %) of individuals who get a diagnosis of lung cancer [8]. This particular form of lung cancer is linked to multiple mutations, involving those that occur in the human epidermal growth factor receptor (EGFR) [[9], [10], [11], [12], [13]]. Tyrosine kinase inhibitors (TKIs) are utilized for the purpose of modulating the human EGFR activity and have emerged as the typical therapeutic approach for individuals afflicted with advanced EGFR-mutant NSCLC. The reversible and competitive binding of first-generation EGFR TKIs, such as gefitinib and erlotinib, occurs at the ATP-binding site situated within the EGFR-TK domain. The present study has demonstrated that this particular interaction has been associated with improved outcomes in individuals diagnosed with NSCLC harboring EGFR-activating mutations, such as L858R and Del 19 mutations [14,15]. However, following an early phase of favourable results, patients experienced a development of resistance to first-generation TKIs as a consequence of the appearance of a T790 M mutation. This mutation specifically influences the ATP-binding domain of the human EGFR residues [[16], [17], [18], [19]].

**Fig. 1:**
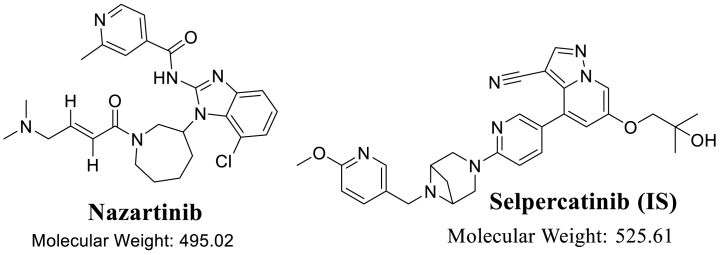
Chemical structures of nazartinib and selpercatinib (IS).

Consequently, the developing of second-generation EGFR TKIs, for example dacomitinib and avitinib, was aimed at specifically targeting cancers harboring both the EGFR-activating mutations and T790 M mutation. These two drugs shown encouraging anti-T790 M efficacy in laboratory-based tests. Nevertheless, the clinical efficacy of these agents against T790M-associated NSCLC was constrained due to their ability to inhibit wild-type EGFR, leading to toxicity and a limited therapeutic range [[20], [21], [22]]. In recent times, the development of third-generation EGFR TKIs, such as nazartinib (NZT) and osimertinib, has taken place. The compounds exhibit irreversible and specific inhibition towards EGFR carrying T790 M and other mutations, while demonstrating minimal impact on the activity of wild-type EGFR [20,21]. Third-generation EGFR TKIs have efficacy against NSCLC which is resistant to both second- and first-generation EGFR TKIs, while also demonstrating little toxicity [23]. Osimertinib, for instance, has obtained approval from both the American regulatory agency, the USFDA, and the European regulatory agency, the European Medicines Agency (EMA), for its utilization in the treatment of patients diagnosed with metastatic EGFR T790 M NSCLC [24]. NZT is under clinical development by Novartis and currently in Phase II for NSCLC [25]. According to pre-clinical evidence, nazartinib (NZT; Fig. 1) has been found to have no impact on the activity of wild-type EGFR and exhibits specificity towards mutant EGFR, comparable to other third-generation EGFR TKIs [26]. However, it is crucial to know that there are certain side effects related to its use, including diarrhea, pruritus, and rash [27].

In a previous study conducted by our research group, we investigated the metabolic processes of NZT, focusing specifically on its bioactivation screening. Through our analysis, we successfully identified the bioactivation center, which is the carbon atom that connects the unsaturated conjugated system and the aliphatic linear tertiary amine. This particular bioactivation center has been implicated as a potential cause for the harmful side effects associated with NZT [28]. In the current research, we assessed the metabolic stability of NZT in vitro employing a UPLC-MS/MS approach. Furthermore, we employed DEREK and P450 software to provide supplementary data. The StarDrop software package, that includes the DEREK and P450 metabolic software, was utilized to evaluate the metabolic stability and possible hazards related to the NZT chemical structure. The investigation of in silico metabolic lability was done employing the P450 program (version 6.6). Metabolic lability pertains to the measurement of the effectiveness of the production phase throughout the catalytic cycle of CYP3A4. The determination of metabolic lability can be achieved through the examination of the composite site lability (CSL) value. A lower CSL designates a greater likelihood of improved stability. The value described above was utilized as first indication to illustrate the importance of implementing practical measures, such as developing analytical methods and conducting in vitro metabolic incubation with HLMs, to adjust time management and resource allocation [29]. The Derek software was employed to conduct a scan for structural alerts inside the NZT chemical structure, in order to provide evidence supporting the proposed idea on in silico metabolic stability. The significance of this study is in the development of an analytical UPLC-MS/MS methodology that possesses several desirable characteristics, including rapidity, environmental sustainability, and high sensitivity. This methodology enables the detection of NZT in various matrices. The precise evaluation of a particular medication (NZT in the current investigation) is essential for the monitoring of therapeutic drug levels (TDM) of said drug. The conducted literature review indicates a scarcity of published scholarly articles pertaining to the determination of NZT in various matrices. To date, there has been no prior scholarly article examining the metabolic stability of NZT in an in vitro experiment employing a UPLC-MS/MS method with the metabolic HLM matrix. In order to estimate the rate of excretion and metabolism, it is important to conduct an analysis of the metabolic stability of NZT in HLMs.

The UPLC-MS/MS system provides superior sensitivity and quicker analysis time compared to HPLC. Additionally, it offers higher sensitivity and reduced interference from endogenous components or metabolites compared to conventional spectrophotometric approaches [[30], [31], [32]]. The UPLC-MS/MS approach employed an isocratic system as the mobile phase, resulting in a short elution time of 1 min indicating an efficient analytical approach. The incorporation of a reduced flow rate at 0.4 mL/min, accompanied by a low concentration of Acetonitrile (ACN), exerted a substantial influence on the advancement of the environmentally friendly attributes of the produced technology. Furthermore, the current methodology exhibited a linearity across a range extending from 1 to 3000 ng/mL. The UPLC-MS/MS technique that was established was assessed for its validity by completing an in silico evaluation of the metabolic stability of NZT utilizing the in silico P450 program, namely StarDrop's software, before doing incubation with HLMs in an in vitro setting [29]. The present study employed this methodology with the goal of dropping expenses and lowering the amount of time required. The current investigation employed the UPLC-MS/MS technique to determine the in vitro half-life (t_1/2_) and intrinsic clearance (Cl_int_) of NZT [33]. The aforementioned methods can be employed to compute the in vivo metabolic rate utilizing three different models, specifically the venous equilibrium, dispersion, and parallel tube models [34,35]. The t_1/2_ and Cl_int_ of NZT were assessed using an in vitro approach based on the well-stirred model [34,35]. The use of this model is commonly used in drug metabolism studies because of its straightforwardness and convenient application [[36], [37], [38]].

## 2 Materials and methods

### 2.1 Materials

HPLC grade solvents were used in the UPLC-MS/MS systems under evaluation. The experiment used analytical grade solid compounds, specifically NZT (Purity: 99.48 %) and selpercatinib (SLP; Purity: 99.87 %). The two analytes under examination, NZT (also known as EGF816) and SLP (also known as LOXO-292), were received from the Princeton, NJ, US-based MedChem Corporation. SLP was used as internal standard (IS) in the quantification of NZT in the HLMs matrix. Formic acid (HCOOH; 98.0–100 %), ammonium formate (NH_4_COOH), dimethyl sulfoxide (DMSO; HPLC, ≥99.7 %), ACN (HPLC Plus, ≥99.9 %), and HLMs were procured from Sigma-Aldrich Firm, a recognized vendor in St. Louis, Missouri, in the US. Dry ice was utilized as a cooling agent during transportation of HLMs to maintain the enzyme viability. From the time of delivery until the time of use, the HLMs (the labelled concentration: 20 mg/mL) matrix were saved at −78 °C in a refrigerator as recommended.

### 2.2 Instruments

The Milli-Q instrument (Millipore Company; Billerica, Massachusetts) was the water filtration system used to produce HPLC-grade water. Acquity UPLC (H10UPH) and Acquity TQD MS (QBB1203), a UPLC-MS/MS instrument, were used as the analytical tools in this work. Following their extraction from the HLMs matrix, the targets (NZT and SLP) were mass analysed using the instrument to identify their analytical peaks. A vacuum pump (the Sogevac Firm; Murrysville, Pennsylvania, USA) was first employed to create the vacuum in the TQD mass analyser. The MassLynx operating program (Version 4.1, SCN 805) was utilized with the UPLC-MS/MS instrument. The QuanLynx program was used to evaluate and translate the results that was gathered. The tuning of MS parameters for NZT and SLP was conducted using the IntelliStart® program. The evaporation of the mobile phase droplets in the ESI source was facilitated by nitrogen gas that was produced by a nitrogen generator (Peak Scientific Firm, based in Scotland, UK). Within the collision cell, the split for NZT and SLP, occurred, generating their corresponding daughter ions. The utilization of argon gas as the collision gas facilitated the acceleration of the fragmentation reaction.

### 2.3 In silico determination of NZT metabolic lability

The in silico metabolic lability of NZT was studied utilizing the StarDrop package, an in silico P450 software established by Optibrium Ltd. in Cambridge, Massachusetts, USA, before conducting in vitro incubation of NZT with HLMs. The efficacy of in vitro experiments was verified employing the data and results obtained from the P450 software. The study's data was subjected to CSL analysis, enabling a comprehensive understanding of the metabolic instability of NZT. Before commencing the in vitro incubation, an in silico determination of the metabolic stability of NZT was conducted utilizing the CSL parameter, which was considered to hold significant significance. The goal of the present study was to assess the feasibility of developing a UPLC-MS/MS method for assessing the metabolic stability of NZT. The use of the SMILES (Cc1cc(ccn1)C(=O)Nc2nc3cccc(c3n2[C@@H]4CCCCN(C4)C(=O)/C=C/CN(C)C)Cl) notation in the metabolic protocol was done to determine the metabolic stability of NZT (CSL). So as to determine the metabolic stability of NZT, the researchers gathered data pertaining to the reactivity of specific atoms, which was subsequently employed to establish the CSL value. Then, this number was employed to acquire comprehension about the metabolic reactivity of NZT [39,40]. The CSL was computed utilizing Equation (1).

(1) CSL=ktotal(ktotal+kw)

where k_w_ is the water formation rate constant.

### 2.4 In silico prediction of the structural alerts of NZT using DEREK module

The evaluation of probable structural alerts for NZT was performed utilizing the DEREK module (the StarDrop package). In addition, the software was utilized to detect structural indications linked to NZT, in order to propose structural modifications that could potentially alleviate the observed toxicity [41,42].

### 2.5 UPLC-MS/MS instrumental features

The analytical settings of the UPLC-MS/MS system were enhanced so as to reach the efficient separation and highest sensitivity of the chromatographic peaks for NZT and SLP. These optimized parameters are outlined in Table 1. Agilent Eclipse plus-C8 column (100.0 mm long, 2.1 mm i.d. and 3.5 μm particle size) at T: 22.0 ± 2.0 °C was used for chromatographic separation of NZT and SLP (IS) peaks. The enhancement of the analytical attributes of the UPLC-MS/MS technique was attained by the careful consideration of several aspects, including the pH level, the mobile phase configuration, and properties of the stationary phase. As listed in Table 1, the target of this optimization was to rise the sensitivity and resolution of the peaks connected to the NZT and SLP. The mobile phase was consisted of 2 distinct lines: line A, that made up of 45 % of the mobile phase and was an aqueous part containing 0.1 % HCOOH in H_2_O at a pH of 3.2; and line B, which made up 55 % of the phase and was an organic solvent made of ACN. The mobile phase flow rate was optimized at 0.4 mL/min. When a 10 mM NH_4_COOH solution was used, tailing in the NZT analytical peak occurred, and the time needed for elution increased when the pH rose above 3.2. Overlapping peaks in NZT and SLP are a notable phenomenon when the concentration of ACN more than 55 %. On the other hand, a lower ACN concentration causes the elution duration to be longer. The nitrogen atoms in the NZT and SLP targets have the ability to absorb protons, causing in the creation of positive-charged ions, hence ionization was made possible by using the positive mode of the ESI source.

**Table 1:** UPLC-MS/MS optimized features.

UPLC conditions		MS/MS (TQD MS) conditions	
Isocratic mobile phase	0.1 % HCOOH in H2O (70 %; pH: 3.2)	ESI	The flow rate of cone gas is 100 L per hour.
	30 % ACN		Positive mode
	Injection volume: 5.0 μl		The voltage of the RF lens is measured to be 0.1 V.
	Flow rate: 0.4 mL/min.		The capillary voltage utilized in this experiment was set at 4 kV (KV).
Eclipse plus-C8 column	100.0 mm long		The voltage of the extractor is 3.0 V.
	2.1 mm i.d.		The drying gas used in this experiment is nitrogen, which is maintained at a temperature of 350 °C. The flow rate of nitrogen is set at 100 L/h.
	3.5 μm particle size	Mode	MRM
	T: 22.0 ± 2.0 °C	Collision cell	Argon gas at 0.14 mL/min

The current research employed the use of SLP as an IS in the determination of NZT. This choice was based on three fundamental variables within the UPLC-MS/MS methodology. The protein precipitation as an extraction method demonstrates its efficacy in extracting both NZT and SLP targets, yielding 100.04 ± 2.71 % (RSD: 2.71) and 100.8 ± 3.14 % (RSD: 3.13 %), respectively. In addition, the chromatographic peaks responding to the target substances NZT (0.49 min) and SLP (0.74 min) were effectively acquired in a time span of 1 min, thereby showcasing the efficacy of the established UPLC-MS/MS technique as a prompt analytical approach, that not only decreases the total duration of the process but also employs a lesser quantity of ACN, thereby adhering to the tenets of green chemistry. Moreover, it is crucial to acknowledge that the patients in a specific medical scenario did not ingest both analytes (NZT and SLP) together. Therefore, the current utilization of the UPLC-MS/MS method is suitable for the purposes of pharmacokinetic and TDM studies of NZT.

The MS tuning of NZT (C_26_H_31_ClN_6_O_2_) and SLP (C_29_H_31_N_7_O_3_) was achieved successfully using the IntelliStart® software and the mutual features of NZT and SLP (10 μg/mL) by direct infusion inside the stream of the running mobile phase. The sensitivity and specificity of the UPLC-MS/MS approach were enhanced by utilizing MRM mode as the best choice for mass analyzer detection approach for the accurate measurement of NZT and SLP. The process of fragmenting NZT and SLP ions into their relevant daughter ions in the collision quadrupole (second quadrupole) was accomplished by utilizing high-quality argon gas. The period of dwell time, in which the transformation of parent ions into fragment ions takes place, is 0.025 s in the context of NZT and SLP. Table 2 exhibits a complete depiction of the various dimensions pertaining to MRM and mass transition in the contexts of NZT and SLP.

**Table 2:** MRM-tuned parameters for the determination of NZT and SLP.

	Time in min.	Rt in min.	Analyte	MRM features
Mass spectra segment	0.0 to 0.65	NZT (0.49)	Analyte: NZT	495 → 112 (CE^a^ 30 and CV^b^ 44)
				495 → 84 (CE: 34 and CV: 44)
	0.65 to 1.0	SLP (0.74)	SLP (IS)	526 → 122 (CV: 42 and CE: 28)
				526 → 53 (CV: 42 and CE: 66)

a Collision energy.

b cone voltage.

### 2.6 NZT and SLP working dilutions

In dimethyl sulfoxide (DMSO), the chemicals NZT and SLP showed the greatest solubility at concentrations of >242 mg/mL (488.87 mM) and 62.5 mg/mL (118.91 mM; ultrasonic assistance was required, correspondingly. Consequently, DMSO was used to dissolve the NZT and SLP stock solutions (1 mg/mL) that were sequentially diluted with the mobile phase to yield working solutions of NZT in μg/mL at 100, 10, and 1 as well as a working solution of SLP at 10 μg/mL.

### 2.7 Establishing of NZT calibration standards

HLMs were inactivated by adding a 2 % DMSO for 5 min at 50 °C prior to carrying out the validation sequence for the UPLC-MS/MS technique. By using this conservative strategy, we hoped to lessen any potential metabolic effects of HLMs [[43], [44], [45]] on the relevant chemicals, namely NZT and SLP. An HLM validation matrix was developed to determine the metabolic stability of NZT. The process involved adding 1 mL of metabolic buffer to 30 μL of HLMs (deactivated) with a protein content of 1 mg/mL. The metabolic buffer contained 1 mM NADPH and 3.3 mM MgCl_2_ and was made up of 0.1 M sodium phosphate with a pH of 7.4. For practical reasons, the goal of this technique was to mimic the environment of an actual in vitro incubation.

So that for establishing calibration standards (CSs) for NZT, a set of dilutions on NZT (WK3 and WK2) were conducted using the HLMs (deactivated). A series of 7 CSs were sequentially prepared, at 1, 5, 15, 200, 500, 1500, and 3000 ng/mL, correspondingly. Furthermore, a set of four distinct quality controls (QCs) with varying concentrations was devised. The lower limit of quantification (LLOQ) values were estimated to be 1 ng/mL, with the lower quality control (LQC) set at 3 ng/mL. The medium quality control (MQC) was established at 900 ng/mL, while the higher quality control (HQC) was set at 2400 ng/mL. So as to alleviate the effect of dilution of matrix and facilitate the replication of in vitro incubates, measures were taken to maintain the ratio of HLMs matrix above 90 % during the dilution procedure. The QCs were used as samples with unknown concentrations, that were assessed by utilizing the regression equation produced from the concurrently introduced NZT CSs. All NZT with a capacity of 100 μL, as well as CSs and QCs, were provided with an IS. This IS consisted of a solution containing SLP WK at 10,000 ng/mL.

### 2.8 Extraction of NZT and PLC from the HLMs matrix

The protein precipitation approach was employed to successfully recover the NZT and SLP analytes from the HLMs matrix. ACN was utilized to successfully neutralize and precipitate the proteins in the HLMs incubation matrix. Consequently, both the NZT CSs and QCs received a 2 mL amount of ACN. So as to enhance the extraction of the required analytes (NZT and SLP) from the HLMs matrix, the mixture was then continuously stirred for 5 min. At 4 °C and speed of 14,000 rpm, centrifugation of the solution took place for the next 12 min. The supernatants were clarified using the centrifugation procedure, which was performed for proteins separation. All incubates underwent a filtration operation using a syringe filter with a pore size of 0.22 μm to prove the dependability and suitability of the samples prepared for injection into the UPLC-MS/MS approach. Before being injected into a UPLC-MS/MS device, the filtered extracts were placed in HPLC vials. For the test, two control samples were created. HLMs made up the first control sample, often known as the negative-control sample. HLMs that had SLP added to them made comprised the second control sample, also known as the positive-control sample. To verify that there was no intervention brought on by the constituents of HLMs through the NZT and SLP processes, the aforementioned techniques were repeated. It was necessary to graph the theoretical standards of NZT on the horizontal axis and the ratio of NZT peak areas to SLP on the vertical axis so as to establish a calibration curve for NZT. By evaluating the linear regression equation (y = ax + b; r^2^) and the UPLC-MS/MS approach's validation parameters, the range of linearity of the constructed NZT CSs was confirmed.

### 2.9 Validation features of the present UPLC-MS/MS approach

The UPLC-MS/MS approach utilized in this work was subjected to validation in line with the requirements prescribed by the US-FDA for the bioanalytical processes validation. The validation approach included the assessment of specific features, including precision, linearity, extraction recovery, stability, sensitivity, specificity, accuracy, and matrix effects [46,47].

#### 2.9.1 Specificity

The evaluation of the UPLC-MS/MS technique specificity necessitated the integration of six distinct sets of blank HLMs samples following to the conclusion of the extraction protocol. The pure extracts were loaded into an UPLC-MS/MS instrument for analysis, with the target of identifying any interfering peaks formed by the matrix that coincided with the elution time of the peaks associated with NZT or SLP. Following this, a comparative analysis was done between the acquired data and the spiked HLMs samples that were improved with NZT and SLP. The implementation of the MRM mode was used to alleviate the carryover influences of NZT and SLP in the TQD quadrupole mass analyzer. The validation of this was achieved by the examination of the findings acquired from the negative control sample HLMs, which were deliberately not subjected to NZT and SLP.

#### 2.9.2 Sensitivity and linearity

The determination of sensitivity and linearity of the UPLC-MS/MS instrument encompassed the injection of twelve calibration curves. The dataset consisted of several curves, each containing seven concentration standards for NZT. The analysis was performed using HLMs as the statistical framework. The examination was conducted within a 24-h period. Following this, the regression equation that was attained from the calibration curve was utilized to ascertain the quantities of NZT present in samples of unidentified concentration. The determination of the LOD and LOQ was performed as outlined in the Pharmacopoeia guidelines. The study's instructions involve determining the LOD and the LOQ utilizing the slope of the SD of the intercept and the linear calibration curve, as outlined in the following Equations (2), (3) respectively.

(2) LOD=3.3*SDoftheinterceptSlope

(3) LOQ=10*SDoftheinterceptSlope

The assessment of linearity in the UPLC-MS/MS approach was done using statistical techniques, specifically the least squared approach (y = ax + b) and the coefficient of variation (r^2^).

#### 2.9.3 Precision and accuracy

The determination of precision and accuracy for the UPLC-MS/MS method entailed conducting numerous experiments spanning three days to measure inter-day analysis, as well as one day for intra-day analysis. So as to conduct inter-day analysis, a total of six sets of NZT QCs were used, but for intra-day analysis, a larger number of twelve sets of NZT QCs were applied. The assessment of the accuracy and precision of the UPLC-MS/MS analytical method consisted of determining the % error (%E) and percent relative SD (%RSD) for quantification purposes. The data were assessed using Equations (4), (5), correspondingly.

(4) %Error=(averagecomputedconc.—supposedconc.)supposedconc.*100

(5) %RSD=SDMean

#### 2.9.4 Extraction recovery and matrix effect

The assessment of the influence of HLMs matrix in the ionization process of the selected analytes, specifically NZT or SLP, was performed by making two separate groups of samples. In the current research study, HLMs were employed to examine the data collected from samples pertaining to group 1. The samples were augmented with the NZT LQC at 3 ng/mL, along with the SLP. In contrast, Group 2 used the mobile phase in place of the HLMs. The estimation of the normalized ME for the IS was done utilizing Equation (6), while the ME for NZT and SLP were estimated utilizing Equation (7).

(6) ISnormalizedME=MEofNZTMEofSLP(IS)

(7) MEofNZTorSLP=meanpeakarearatioGroup1Group2×100

This research focused on evaluating the process of extracting NZT from the HLMs matrix and investigating the influence of HLMs on the extent of NZT parent ionization. The accomplishment was attained by providing four QC samples by injection. The evaluation of protein precipitation as an extraction methodology for NZT and SLP encompassed the utilization of six cohorts, each including four QC samples inside a matrix composed of HLMs (B). Subsequently, the aforementioned samples were compared with four QC samples that were formulated using the mobile phase (A). The extraction recoveries for NZT and SLP were estimated by computing the ratio of B to A, which was subsequently multiplied by 100.

#### 2.9.5 Stability

The assessment of NZT's stability in HLMs matrix and stock solutions was performed in different laboratory settings, encompassing pre-analysis protocols such as auto sampler storage, long-term storage and short -term storage, and three thaw-freeze cycles.

### 2.10 In vitro determination of the NZT metabolic stability

The assessment of Cl_int_ and the calculation of the in vitro t_1/2_ of NZT (the chemical being studied) required the analysis of the remaining amount of NZT after its exposure to an in vitro incubation. The incubation methodology involved the utilization of a dynamic preparation of HLMs, that contained NADPH as a cofactor and MgCl_2_. The in vitro incubation was employed utilizing a four-step approach. During the first phase, a 1 μL quantity of NZT was pre-incubated with a matrix composed of metabolic HLMs. The aforementioned approach was performed within a water bath that underwent exact temperature control at 37 °C for 10 min. At the commencement of the experiment, 1 mM NADPH was added to each individual sample. Following this, the entirety of the samples was reintroduced into a thermostatic shaking water bath, whereby the temperature was adjusted at 37 °C. In the third phase of the experiment, the procedure for introducing the sample entailed dispensing 100 μL of SLP (1000 ng/mL) into the system, which was subsequently followed by the application of ACN as a searing agent. The aim of this approach was to attain a consistent peak intensity of the IS and mitigate any possible influence of the metabolism on the IS concentration. During the final stage, the introduction of 2 mL of ACN at designated points (0, 2.5, 7.5, 15, 20, 30, 40, 50, 60, and 70 min) was executed to cease the metabolic processes and initiate the sedimentation of excess proteins. The first step of the extraction process for NZT and SLP is considered to be the initial stage, as described in Section 2.9. A negative control experiment was employed to explore the effects of NZT on HLMs in the absence of NADPH, using the methods described before. The objective of the present study was to determine the potential influence of incubation settings and matrix effects on the concentrations of NZT in in vitro incubation tests.

The quantification of the remaining NZT was conducted by employing the regression equation obtained from the concurrent injection of NZT CSs. The construction of the metabolic stability curve for NZT included the utilization of a specific approach. This technique involved the graphical depiction of specific time intervals from 0 to 70 min, on the x-axis. As well, the equivalent % of NZT concentration that remained in relation to the original concentration at zero time (100 %) was represented on the y-axis. Consequently, the logarithmic curve was derived by specifically examining the portion of the metabolic curve from 0 to 40 min. The previously indicated range was employed to graph the natural logarithm (ln) of NZT concentrations against their corresponding metabolic periods. The assessment of the rate constant for NZT metabolic stability can be accomplished by assessing the gradient of the aforementioned graph. The slope that was determined through calculations was subsequently employed to estimate the t_1/2_ in an in vitro environment, employing the formula in vitro t_1/2_ = ln2/slope. The NZT Cl_in_t (mL/min/Kg) was determined utilizing the ratio of liver tissue mass (26 g) per kg of body weight to the mass of the HLMs (45 mg) per gram of liver tissue, as previously reported in studies (Equation (8)) [48,49].

(8) $${\text{Cl}}_{\mathrm{int},}=0693\;x\;\frac{1}{{\;t\;}_{½}\left(\min.\right)}\times \frac{\text{mL}\;\text{incubation}}{\text{mg}\;\text{protein}}\times \frac{\text{mg}\;\text{microsomal}\;\text{proteins}}{g\;\text{of}\;\text{liver}\;\text{weight}}\times \frac{g\;\text{liver}}{\text{Kg}\;b.\;w.}$$

## 3 Results and discussions

### 3.1 In silico P450 metabolic lability of NZT

**Fig. 2:**
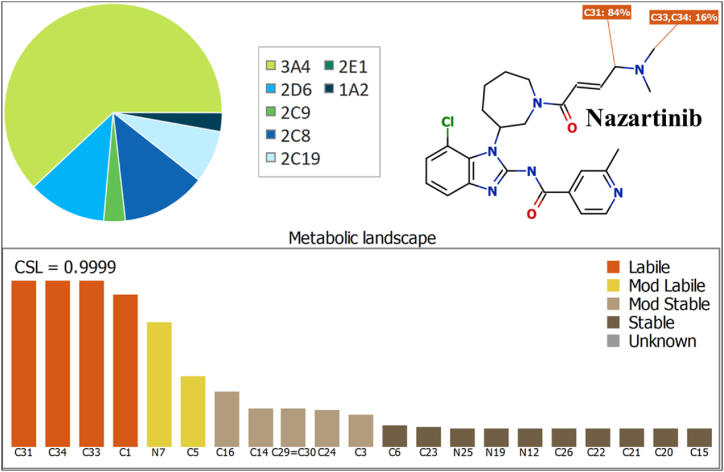
The CSL (0.9999) demonstrates the significant susceptibility of NZT to metabolic processes. The assessment of the outcomes was done using WhichP450 (the StarDrop software package).

The metabolic profile of NZT was used to make assumptions concerning the vulnerability of active areas in the chemical structure of NZT to enzymatic effects by the CYP3A4 enzyme. The data was represented in a visual style using a pie chart [[50], [51], [52]]. The CSL value of 0.9999 (Fig. 2) provided evidence for the significant metabolic instability of NZT. The UPLC-MS/MS methodology was utilized to test the metabolic stability of NZT after metabolic incubation with HLMs. The metabolic stability was seen to be significant at carbon C33 and C34 (16 %) of the dimethylamino group, C31 (84 %) of the butenoyl group, and C1 (1 %) of the isonicotinamide group. The metabolism of NZT was investigated, revealing that the carbon atom (C31) linking the aliphatic linear tertiary amine and the unsaturated conjugated system undergoes bioactivation. The outcomes of this study align with a previous inquiry into the metabolic processes associated with NZT [28], which shown that C31 of the butenoyl group accounts for 84 % of the metabolic instabilities of NZT (Fig. 2; CSL: 0.9999, showing a significant vulnerability to metabolism). The aforementioned results align with the data of the in vitro metabolic tests, that will be illustrated upon in the next sections.

### 3.2 In silico DEREK screening for NZT structural alerts

**Fig. 3:**
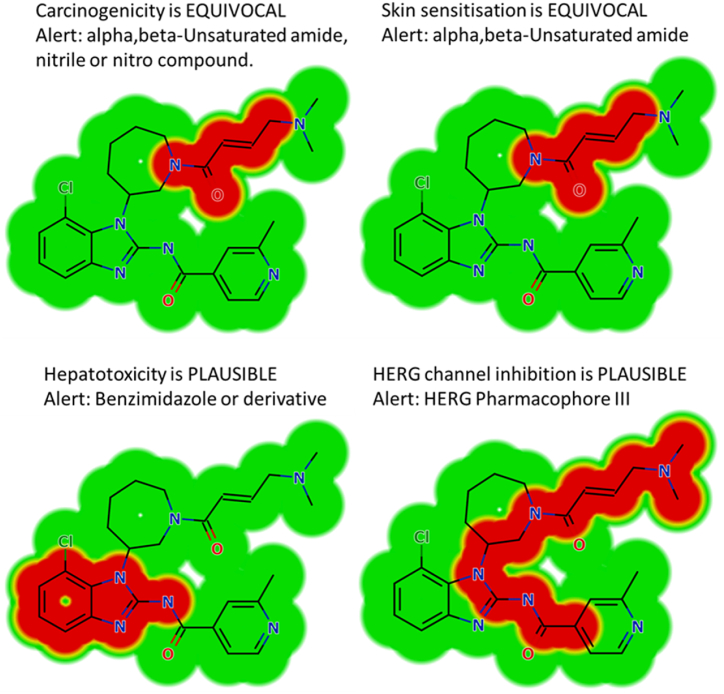
DEREK software results showing structural alerts of NZT chemical structure marked in red colour. (For interpretation of the references to color in this figure legend, the reader is referred to the Web version of this article.)

The in silico toxicity determination of NZT was made employing the DEREK software, as depicted in Fig. 3. The NZT demonstrates structural alerts, as depicted in Fig. 3. The aforementioned notifications are accountable for the postulated consequences, specifically the potential for carcinogenicity (with equivocal evidence) attributed to compounds containing alpha, beta-unsaturated amide, nitrile, or nitro groups. Additionally, there is equivocal evidence suggesting that skin sensitization may occur due to the presence of alpha, beta-unsaturated amide compounds. Furthermore, there is a plausible association between the inhibition of HERG channels (via HERG Pharmacophore III) and these alerts. Lastly, the plausible connection between hepatotoxicity and benzimidazole or its derivatives is also worth noting. The three potential side effects, namely carcinogenicity, skin sensitization, and HERG channel blockage, are linked to the dimethylamino and butenoyl groups that are interconnected. The preceding findings have demonstrated that the observed toxicity and metabolic instability can be attributed to the presence of the dimethylamino and butenoyl functional groups, which aligns with the prior outcomes attained from metabolic bioactivation studies and in silico assessments of metabolic stability. Modifications to the bioactive groups, namely the dimethylamino and butenoyl groups, as well as replacement of the group in the context of drug design, possess the capacity to augment the safety and metabolic stability characteristics of novel derivatives when compared to NZT (Fig. 3).

### 3.3 UPLC-MS/MS method

A variety of stationary phases were examined, including columns utilizing hydrophilic interaction liquid chromatography (HILIC). Both the NZT and SLP chemicals did not demonstrate any retention or resolution. The use of a C8 column as the reversed stationary phase has shown favourable results. However, while employing a C18 column in the UPLC-MS/MS approach for the resolution of NZT and SLP that were found to be retained during the chromatographic process. Nevertheless, it is evident that NZT and SLP displayed suboptimal differentiation of the primary peak, asymmetry in the form of the peak, and a lengthier retention time. The utilization of an Eclipse plus-C8 column (3.5 μm, 2.1 mm, and 100 mm) generated favourable results in relation to peak shape and retention time. In the current approach utilizing UPLC-MS/MS, the chemicals NZT and SLP were chromatographically separated by applying an isocratic mobile phase with a flow rate at 0.4 mL/min for a 1 min. The calibration curve for NZT, as created using the described methods, exhibited a linear correlation from 1 to 3000 ng/mL. Table 3 provides data of various studies employed to determine and enhance the optimal attributes for the extraction, separation, and assessment of NZT and SLP peaks. The primary objective of these investigations was to attain favourable attributes, such as a well-defined analytical peak form and decreased retention time.

**Table 3:** Tuned parameters of the current UPLC-MS/MS technique for NZT and SLP.

	Extraction Recovery		Mobile Phase		Stationary Phase	
	Protein Precipitation Using ACN	Solid Phase Extraction	ACN	Methanol	C8 Column	C18 Column
NZT	High (100.04 ± 2.71 %)	Low (85.32 %)	0.49 min	0.56 min	0.49 min	1.12 min
	Precise (RSD <2.71 %)	Not precise	Optimum peak	Tailed	Good shape	Tailed peaks
SLP	High (99.55 ± 3.92 and)	Low (68.17 %)	0.74 min	0.85 min	0.74 min	1.32 min
	Precise (RSD <3.93 %)	Not precise	Optimum peak shape	Overlapped	Perfect shape	Perfect shape

**Fig. 4:**
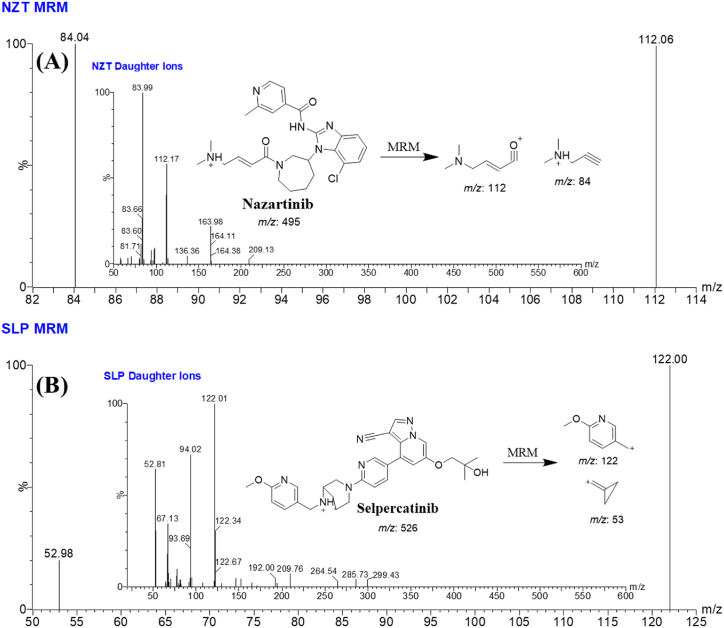
MRM mass spectrum of NZT [M+H]+ showing daughter ions scan (A) and SLP (B) [M+H]+ showing daughter ions scan. The observed fragmentation patterns are demonstrated.

So as to elevate the sensitivity of the present approach, the application of the MRM analyzer as the best of choice detection mode was performed to enable the estimation and detection of NZT and SLP. The aim of this experiment was to address and decrease any probable disruptions produced by the matrix ingredients present in the HLMs (Fig. 4).

**Fig. 5:**
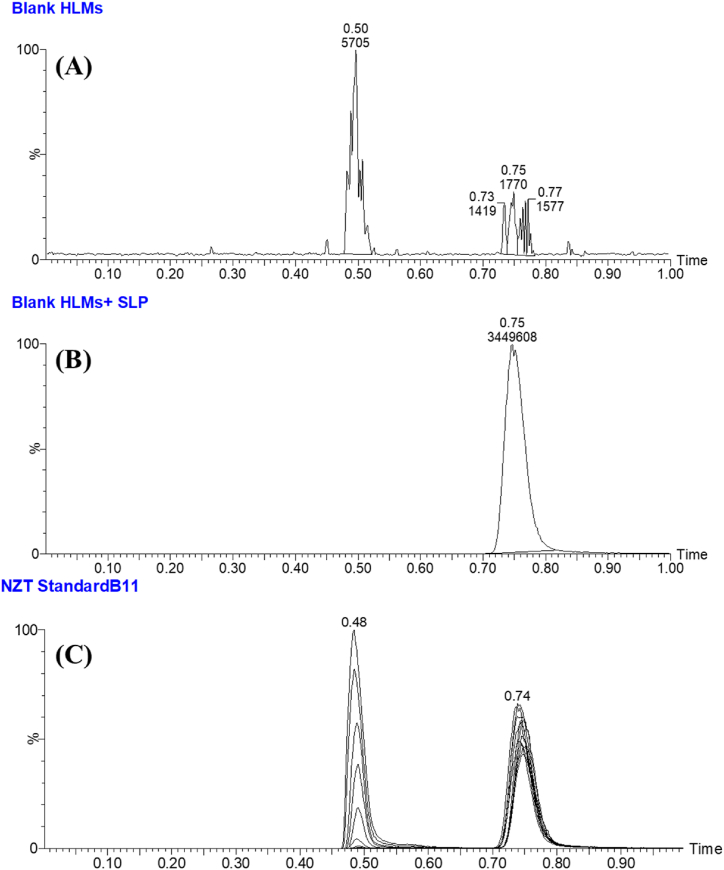
(A) HLMs matrix (Negative-control) exhibiting small carry over effect of NZT and SLP (IS) at the triple quadrupole mass analyzer; **(B)** MRM chromatogram of positive-control (Blank HLMs plus SLP; 1000 ng/mL). **(C)** Overlaid MRM chromatograms of NZT QCs and CSs revealing the NZT chromatographic peaks (0.48 min) and SLP chromatographic peaks (1000 ng/mL; 0.74 min).

The MRM mass chromatograms of the positive control samples (Fig. 5B) and negative control samples (Fig. 5A) for NZT exhibited minimal carry-over influence. Fig. 4C presents the overlaid MRM chromatograms of NZT QCs and CSs revealing the NZT chromatographic peaks (0.48 min) and SLP chromatographic peaks (1000 ng/mL; 0.74 min).

### 3.4 Validation of the UPLC-MS/MS method

#### 3.4.1 Specificity

The UPLC-MS/MS technology's validity was determined by effectively separating chromatographic peaks associated with NZT and SLP, as shown in Fig. 5. Additionally, it was noticed that the peaks for NZT and SLP, did not show any notable interventions from the matrix constituents found in the HLMs incubation matrix. The mass chromatograms attained from the control samples during the MRM study did not reveal any discernible impacts attributed to carry-over effects emanating from NZT.

#### 3.4.2 Sensitivity and linearity

**Fig. 6:**
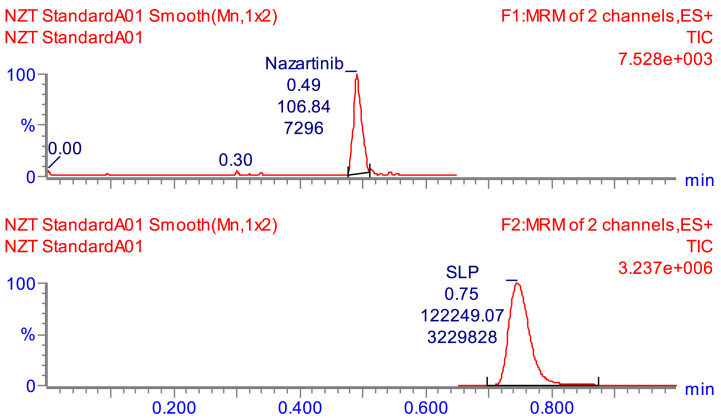
The sensitivity of the current UPLC-MS/MS methodology is exemplified by its ability to estimate the NZT analytical peak at 1 ng/mL (**A**), that resembles the LOQ. Furthermore, the analytical peak of the SLP is detected at 1000 ng/mL (**B**).

The statistical approval of the linearity of the UPLC-MS/MS method was performed across the range from 1 to 3000 ng/mL. The achievement was obtained by incorporating NZT CSs into the HLMs and then inferring the outcomes as variables. The confirmation of data linearity was achieved by the application of regression analysis, resulting in the derivation of the equation y = 0.3724x + 0.2749. The coefficient of determination (R^2^) in this linear model was evaluated to be 0.9936. So as to account for the wide range of concentration values exhibited by the CSs, a weighting feature of (1/x) was utilized during the graphing of the NZT calibration curve. The examination of the data provided in Table 4 indicated that the RSD of the 6 repeats, encompassing CSs, was observed to be below 7.10 %. The LOD and LOQ were determined at 0.13 ng/mL and 0.39 ng/mL, correspondingly, as showed in Fig. 6.

**Table 4:** Back-calculation results of 6 repeats of NZT CSs.

NZT (ng/mL)	Mean	SD	RSD (%)	Accuracy (%)	Recovery
1	1.07	0.08	7.10	6.67	106.67
5	4.90	0.08	1.56	−1.93	98.07
15	15.07	0.29	1.91	0.49	100.49
50	50.48	2.57	5.09	0.97	100.97
200	192.90	5.64	2.93	−3.55	96.45
500	503.09	3.82	0.76	0.62	100.62
1500	1486.87	25.07	1.69	−0.88	99.12
3000	2971.18	18.58	0.63	−0.96	99.04
% Recovery					100.04 ± 2.71

#### 3.4.3 Precision and accuracy

The assessment of precision and accuracy for the UPLC-MS/MS approach was done by the implementation of 12 experimental sets, wherein each set comprised four QCs. The experiments were conducted throughout a 24-h period. Following that, a total of 6 sets, each involving four QCs, were carried out consecutively throughout the course of the subsequent three-day period. The acquired results were found to be within the permissible range defined in the validation principles outlined by the FDA [53]. The UPLC-MS/MS approach which was demonstrated diverse accuracy and precision metrics on several days, as detailed in Table 5. The inter-day accuracy and precision revealed a variability of values ranging from −4.33 % to 4.43 %. Similarly, the intra-day accuracy and precision displayed a range from −2.78 % to 7.10 %.

**Table 5:** The present approach of UPLC-MS/MS exhibits characteristics pertaining to the validation of preci-sion and accuracy.

Statistical parameters	Intra-Day (12 Sets in 1 Day)				Inter-Day (6 Sets in 3 Days)			
QCs	1.00	3.00	900	2400	1.00	3.00	900.00	2400.00
Mean	1.07	2.92	907.08	2425.45	0.96	3.04	889.65	2392.16
SD	0.08	0.03	7.60	6.55	0.02	0.13	6.54	6.68
Precision (%RSD)	7.10	0.86	0.84	0.27	2.18	4.43	0.74	0.28
% Accuracy	6.67	−2.78	0.79	1.06	−4.33	1.33	−1.15	−0.33
Recovery (%)	106.67	97.22	100.79	101.06	95.67	101.33	98.85	99.67

#### 3.4.4 The utilization of HLMs matrix does not exert an influence on the process of extraction recovery of NZT inside the UPLC-MS/MS instrument

The efficiency of the UPLC-MS/MS approach employed for the protein precipitation as the selected extraction technique of NZT and SLP was assessed through the performance of six repeated analyses, which included four QCs. These analyses were conducted utilizing the HLMs matrix. Then, the data were compared to QCs that were prepared using the mobile phase. The study's findings revealed a significant degree of extraction recovery for NZT (100.04 ± 2.71 % and RSD <2.71 %) and SLP (99.55 ± 3.92 and RSD <3.93 %). The analysis of two groups of loaded samples of HLMs has substantiated that it does not exert any impact on the ion formation process, specifically pertaining to the ions NZT or SLP. In the experimental configuration, Group set 1 was exposed to the introduction of NZT LQC (3 ng/mL) and SLP (1000 ng/mL). In contrast, sample group 2 was produced through the substitution of the HLMs with the mobile phase. HLMs samples that utilize NZT and SLP had a ME of 100.41 ± 3.43 % and 99.67 ± 2.04 %, respectively. The normalized ME for the IS was 1.02, that is within the allowed range stated by the rules defined by the FDA. The findings indicate that the HLMs matrix doesn't exert a statistically noteworthy influence on the extent of parent ionization for both SLP and NZT.

#### 3.4.5 The NZT exhibited stability when introduced into the DMSO and HLMs matrix

The calculation of the stability of NZT in DMSO and the HLMs matrix, shown that best stability was accomplished by storing NZT in DMSO at −80 °C for a period of 28 days. The RSD% of all NZT samples was determined to be below 2.22 % through all storage circumstances, as listed in Table 6. There was no observed substantial reduction in the NZT concentration subsequent to brief-term storage, storage in an auto-sampler, three cycles of thawing and freezing, as well as extended-term storage. The findings presented in this study offer substantiation for the substantial level of stability exhibited by NZT.

**Table 6:** NZT stability parameters.

Stability Parameters	3.0	2400.0	3.0	2400.0	3.0	2400.0	3.0	2400.0
	Mean		SD		RSD (%)		Accuracy (%)	
Auto-Sampler Stability (24 h at 15 °C)	3.01	2399.30	0.04	12.65	1.34	0.53	0.44	−0.03
Long-Term Stability (−80 °C for 28 d)	2.95	2395.02	0.07	9.62	2.22	0.40	−1.67	−0.21
Freeze–Thaw Stability (−80 °C for 3 cycles)	2.91	2387.65	0.05	3.02	1.72	0.13	−3.00	−0.51
Short-Term Stability (room temperature for 4 h)	3.04	2400.27	0.04	11.66	1.37	0.49	1.22	0.01

### 3.5 An evaluation of the environmental greenness of the UPLC-MS/MS method with AGREE software

**Fig. 7:**
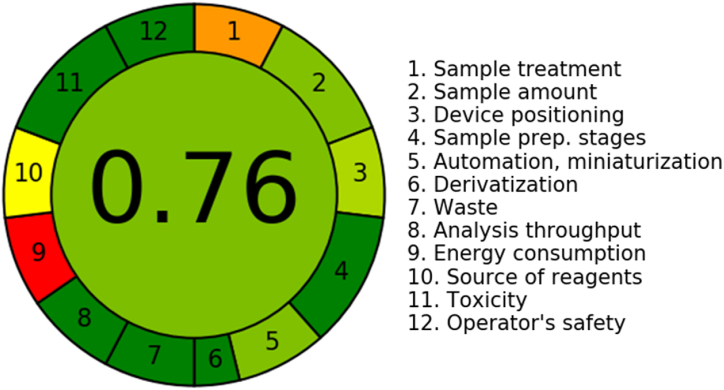
The results are graphically shown using a circular diagram, that showcases a diverse spectrum of colors ranging from red (indicating absence of greenness) to dark green (indicating the maximum degree of greenness). The colors listed above are related to 12 separate features, as illustrated in the accompanying diagram. (For interpretation of the references to color in this figure legend, the reader is referred to the Web version of this article.)

The determination of the sustainability also stated as the "greenness," of the proposed UPLC-MS/MS methodology was performed utilizing the AGREE software tool, which operates inside a statistical framework. The software incorporates the 12 parameters specified by the GAC initiative [54]. The software uses a variety of weights spanning from 0.0 to 1.0 to allocate values to certain features of the GAC system. This methodology enables the developing of analytical scales that successfully assess the degree of ecological sustainability. The outcomes are exhibited by a circular arrangement that incorporates a wide range of colors, spanning from red to dark green, with each color symbolizing twelve unique characteristics. Fig. 7 illustrates the present application of UPLC-MS/MS technology within the framework of its environmental sustainability. The results pertaining to all 12 attributes were recorded and shown in Table 7. The numerical value of 0.76 was ascertained by a comprehensive assessment of many elements pertaining to the present technique. The grade helps as a quantitative estimate for the sustainability degree that is attained using the UPLC-MS/MS technology. It is essential to know that a higher score (near 1.0) means an increased level of ecological sustainability in the assessment process. The present UPLC-MS/MS system demonstrates a distinguished degree of sustainable development, as approved by eco-scale scores (0.75–1.00).

**Table 7:** The report sheet for the UPLC-MS/MS method has been established to evaluate the environmental greenness.

Criteria	Score	Weight
1. It is desirable to use direct analytical methods so as to reduce the need of sample preparation.	0.3	2
2. The primary goals of this study are to achieve reduction in the quantity of samples and a limited sample size.	0.75	3
3. Preferably, it is advisable to conduct assessments in their initial contextual framework.	0.66	
4. The combination of analytical methodologies and operational practices has been found to yield energy saving benefits and a reduction in reagent consumption.	1.0	2
5. It is advisable to go for automated and reduced procedures.	0.75	3
6. It is desirable to refrain from using derivatization approaches.	1.0	2
7. Minimizing the formation of considerable quantities of analytical waste and implementing effective trash management procedures are crucial.	1.0	1
8. There is a preference for utilizing multi-parameter or multi-analyte approaches instead of depending just on single-analyte procedures.	1.0	2
9. It is imperative to exert endeavors aimed at reducing energy use.	0.0	2
10. It is desirable to prioritize the consumption of reagents resulting from sustainable sources.	0.5	2
11. The elimination or substitution of dangerous substances is of utmost importance.	1.0	3
12. There is a compelling need to enhance the safety protocols for employees.	1.0	3

### 3.6 In vitro incubations of NZT with HLMs matrix

**Fig. 8:**
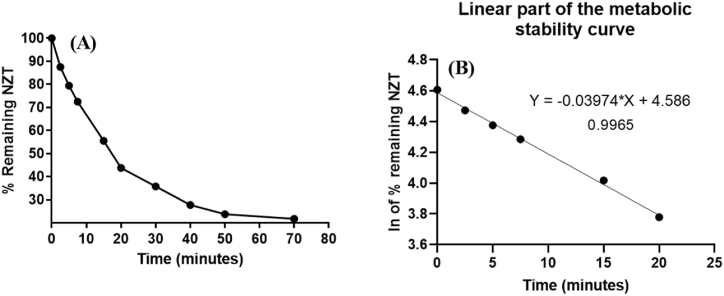
The NZT metabolic stability curve (**A**). The linear part of the logarithm (ln) curve showing the linear regression equation that was employed to assess the NZT metabolism rate (**B**).

NZT concentration. To determine the metabolic stability of NZT (1 μM/mL), an in vitro approach was utilized. This study employed HLMs for carrying out metabolic incubation experiments. The choice of this precise concentration was driven by the aim of maintaining the concentration beneath the Michaelis-Menten constant. This approach ensures the maintenance of a direct correlation between the rate of NZT metabolism and the length of the in vitro metabolic incubation period. To avoid the matter of non-specific protein binding, HLMs (1 mg of protein) in each mL of incubation buffer were utilized. The creation of the primary metabolic stability curve for NZT entails the depiction of specific temporal pauses for annealing from 0 to 70 min along the x-axis. The vertical axis in Fig. 8A represents the residual ratio of NZT. The linear portion of the previous curve was chosen within the time interval of 0–40 min so as to produce an additional curve that illustrates the relationship between incubation time (0–40 min) and the natural logarithm of the residual ratio of NZT (Fig. 8B). The investigation's findings indicate that the metabolic rate of NZT, as represented by the slope, was found to be 0.03974. The equation y = −0.03974x + 4.586 was employed to figure out this data, and the r^2^ was found to be 0.9965, as shown in Table 8. The estimation of the in vitro t_1/2_ can be accomplished by employing the formula ln2/slope. The t_1/2_ value observed in vitro for this specific case was determined to be 17.44 min. The NZT Clint was determined to have a value of 46.49 mL/min/kg. According to McNaney et al., NZT is categorized as a pharmaceutical characterized by its rapid clearance capabilities. This classification implies that the administration of NZT does not present a potential risk of accumulating doses within the human body. The utilization of software tools, for example Cloe PK and modelling software, has the capacity to provide significant insights into the in vivo pharmacokinetics of NZT, hence uncovering essential physiological aspects.

**Table 8:** Metabolic stability of NZT.

Time (min.)	Mean^a^ (ng/mL)	X^b^	LN X	Linearity parameters
0.00	465.75	100.00	4.61	Regression line equation: y = −0.03974x+4.586
2.50	407.48	87.49	4.47	
5.00	369.90	79.42	4.37	R^2^ = 0.9965
7.50	337.62	72.49	4.28	
15.00	258.40	55.48	4.02	Slope: −0.03974
20.00	203.77	43.75	3.78	
30.00	166.46	35.74	3.58	t_1/2_: 17.44 min and
40.00	129.39	27.78	3.32	Cl_int_: 46.49 mL/min/kg
50.00	110.76	23.78	3.17	
70.00	101.25	21.74	3.08	

a Mean of three replicates.

b X: Average of the % of residual concentration of NZT in three repeats.

The negative-control sample displayed no noteworthy decrease in the level of NZT. An in vitro technique was employed to assess the metabolic stability of NZT at a concentration of 1 μM/mL. The present work utilized HLMs to conduct metabolic incubation studies. The decision to use this specific concentration was motivated by the objective of keeping the concentration below the Michaelis-Menten constant. This approach guarantees the preservation of a direct association amongst the rate of NZT metabolism and the time specified for the in vitro metabolic incubation. In order to alleviate the problem of non-specific protein binding, HLMs at 1 mg/mL was employed. The process of constructing the basic metabolic stability curve for NZT involves representing distinct time intervals for annealing, which span from 0 to 70 min, on the x-axis. The residual ratio of NZT is represented on the vertical axis in Fig. 8A. The selection of the linear part in the earlier graph was made specifically for the time range of 0–40 min. This was done to generate an additional graph that depicts the correlation between incubation time points (ranging from 0 to 40 min) and the natural logarithm of the residual ratio of NZT, as shown in Fig. 8B. The results of the experiment reveal that the metabolic rate of NZT, as denoted by the slope, was estimated to be 0.03974. The equation y = −0.03974x + 4.586 was employed to calculate the aforementioned data, and the r^2^ was determined to be 0.9965, as listed in Table 8. The calculation of the in vitro t_1/2_ can be accomplished by utilizing the formula ln2/slope. The t_1/2_ value measured in an in vitro setting for this particular case was found to be 17.44 min. The NZT Cl_int_ was found to possess a value of 46.49 mL/min/kg. According to McNaney et al. [45], NZT is classified as a medication known for its efficient clearance properties. This classification suggests that the administration of NZT does not provide a potential risk of dosage accumulation in the human body. The application of software tools, such as modeling software and Cloe PK, has the potential to offer valuable insights into the in vivo pharmacokinetics of NZT, hence revealing crucial physiological characteristics [55].

## 4 Conclusions

**Fig. 9:**
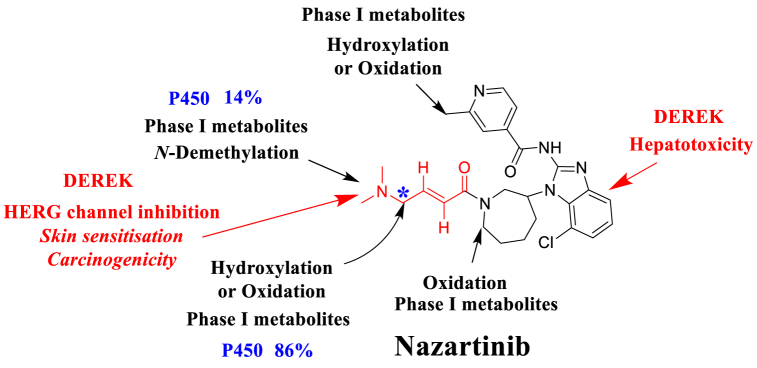
(NZT metabolic lability score (blue color), NZT DEREK toxicity predictions (red color) and NZT phase I metabolites (black color) revealing that dimethylamino and the butenoyl groups are accountable for the metabolic instability of NZT. (For interpretation of the references to color in this figure legend, the reader is referred to the Web version of this article.)

The primary objective of this research is to develop a rapid and efficient UPLC-MS/MS approach for the assessment of NZT in the HLMs matrix. Next, the aforementioned methodology was used to determine the NZT metabolic stability. The application of protein precipitation as the selected extraction method in the UPLC-MS/MS approach has exhibited numerous beneficial qualities, such as enhanced selectivity, sensitivity, environmental compatibility, and efficient recovery of NZT and SLP from the HLMs matrix. The UPLC-MS/MS technology used in the present study showed its environmentally friendly attributes through the integration of diverse methodologies. The aforementioned modifications encompassed the usage of a diminished flow rate at 0.4 mL/min, a lower concentration of ACN (55 %), and a condensed analysis time for 1 min. Through an evaluation of the method greenness using the AGREE programs, it can be assumed that the current UPLC-MS/MS approach has desirable ecological characteristics and could be used for the routine measurement of NZT without causing side effects on the nearby ecosystem. The confirmation of the validity of computational analysis of P450 metabolism using StarDrop's software was done through performing in vitro metabolic incubation tests with HLMs. The data obtained on the metabolic stability of the NZT compound reveals a t_1/2_ of 17.44 min and a moderate Cl_int_ of 46.49 mL/min/kg. The findings of this investigation suggest that NZT demonstrates attributes consistent with those of a pharmaceutical agent possessing a high extraction ratio. Hence, it is postulated that the administration of NZT may be a viable option for patients, given its purported minimal tendency for dose accumulation inside the human organs. Future research efforts may include the application of computational software and experimental metabolic incubations, that are crucial in the improvement of metabolic stability of the new developed medications. The effectiveness of in silico software in enhancing resource allocation and minimizing labor can be demonstrated by comparing the outcomes derived from in vitro metabolic tests with the study of NZT using in silico software. Depending on the data obtained from in silico analyses using DEREK and P450 metabolic software, it is advisable to consider making minor structural modifications to the dimethylamino butenoyl moiety or substituting the cited group at the drug design phase. This approach may offer a potential means to increase the safety and metabolic stability characteristics of novel derivatives, if compared to NZT (Fig. 9). The application of these approaches is crucial for developing of innovative pharmaceuticals, chiefly in enhancing metabolic stability.

## Ethics approval

The usage of HLMs procured from the Sigma company releases it from the requirement of getting ethical approval.

## Funding

This study was funded by the Researcher Supporting Project Number (RSPD2024R760), 10.13039/501100002383King Saud University, Riyadh, Saudi Arabia**.**

## Data availability statement

All data are available within the manuscript.

## CRediT authorship contribution statement

**Mohamed W. Attwa:** Writing – review & editing, Writing – original draft, Validation, Resources, Methodology, Investigation, Funding acquisition, Conceptualization. **Ali S. Abdelhameed:** Writing – review & editing, Supervision, Resources, Project administration, Investigation, Formal analysis. **Adnan A. Kadi:** Writing – review & editing, Visualization, Supervision, Project administration, Funding acquisition, Formal analysis, Data curation.

## Declaration of competing interest

The authors declare that they have no known competing financial interests or personal relationships that could have appeared to influence the work reported in this paper.
